# Precision Regulation Approach: A COVID-19 Triggered Regulatory Drive in South Korea

**DOI:** 10.3389/fpubh.2021.628073

**Published:** 2021-02-01

**Authors:** Sora Lee, Woojin Kang

**Affiliations:** ^1^Menzies Centre for Health Governance, School of Regulation and Global Governance (Regulatory Network), ANU College of Asia & the Pacific, The Australian National University, Canberra, ACT, Australia; ^2^Department of Economics, College of Economics and Business Administration, Hanbat National University, Daejeon, South Korea

**Keywords:** COVID-19, precision regulation, deregulation, biotech industry, South Korea

## Abstract

COVID-19 has triggered various changes in our everyday lives and how we conceptualize the functions of governments. Some areas require stricter forms of regulation while others call for deregulation. The challenge for the regulatory authorities is to manage these potentially conflicting demands in regulation and define coherently their overall regulatory rationale. The precision regulation approach can be a helpful approach. It is defined here as a streamlined approach to regulation to deliver the right methods of regulation for the right group of people at the right time. This problem-solving innovation in regulation triggered by the recent epidemiologic crisis in South Korea demonstrates the emergence of the precision regulation approach. South Korea has implemented streamlined fast-track services for the biotechnology industry to produce test kits swiftly. This article expands the definition of precision regulation from AI regulation literature, and positions the term as a new regulatory rationale, not as a regulatory tool, using the case study from South Korea.

## Introduction

Crises drive various changes in our lives. COVID-19 is a global pandemic with 48.5 million cases, and 1,231,017 death confirmed worldwide as of November 6th, 2020 (1). The confining responses of COVID-19, such as lockdowns, quarantine, and self-isolation, have significantly disrupted how we live, work, study, and travel and challenge the norms of what constitutes normality (2). Beyond the everyday routine, the pandemic has broadly impacted legislative reforms and deregulation agendas worldwide; the nations strived to adapt to the needs of government, industry, and civil society under the COVID-19. Regulatory amendements are occurring beyond the medical system to cope with COVID-19 and the post-COVID-19 era.

The level of regulation slides on a binary scale of the regulatory flexibility. The extremities of the binary distinction in regulation, however, may cause public services to be controlled in markets or, conversely, move toward a paternalistic “big brother state” (3). Furthermore, post-pandemic transformations are still unfolding fast and remain uncertain (4). The binary conception of regulation does not leave much room for regulators to adjust after the number of cases dropped. This article highlights an example set by South Koreanbiotechnology industry regulation to illustrate a precision regulation approach, an emergeant regulatory approach to break the binary distinction by combining the deregulation with careful scrutiny. This article aims to expand the concept of precision regulation, which is only applied in technology regulations at present. There may be benefits for the term to be positioned as a new regulatory rationale beyond a regulatory tool.

## The Precision Regulation Approach

The precision regulation approach can thus be defined here as a problem-solving approach to regulation to deliver the right methods of regulation for the right group of people at the right time. It has originated from the term “precision medicine,” which is a model that proposes the customization of healthcare, with medical decisions, treatments, practices, or products being tailored to a subgroup of patients, instead of an one-drug-fits-all model (5). In medicine, precision medicine is defined as a healthcare that is finely tuned to each individual. Properly implemented, it has the potential to shift the focus of the health system from the treatment of illness to the protection of health (6). The scholars in the area of precision medicine regulation explicitly claim that “the sector needs to remain adaptive, flexible, and responsive” [(7), p. 299]. Thus, Nicole et al. (7) further suggest that the regulation of such medical model should be based on appropriate consideration of safety, efficacy, cost effectiveness, consistency across geographical, technological and institutional borders, cultural respect, and inclusiveness.

The term “precision regulation” has taken off the field of medicine to be applied to other realms of regulation. IBM Policy Lab also mentions the precision regulation approach for technology in January 2020 to suggest an alternative framework to regulate companies in creating, distributing, or commercializing AI systems (8). The framework details five steps to have trustworthy AI: nurturing an AI ethics specialist; applying an individualized approach to risks, promote transparency among stakeholders; contextualize AI and communicate with regulators; and test for fairness and bias. While the above regulatory rules may be specific to regulating the AI technology, this problem-solving regulatory approach resonates with sectors and governments beyond the AI. Such an application of precision regulation in AI suggest that perhaps the term can be potentially useful for regulators who struggle to find the right balance of regulations post-COVID-19.

## Binary Scale of Regulation: From Stricter Regulation to Deregulation

The current conceptualization of regulation rests mainly on binary perception. Facing the direct risks of COVID-19, strict spatial confinements were a prominent feature of the earlier phase of the pandemic. As a response to this global disease threat, strict movement restrictions and the travel ban were placed in and out of Wuhan on January 23rd, 2020 (9). Neighboring countries, Hong Kong, Singapore, and South Korea, quickly followed the response, suppressing the disease successfully compared to other countries (10). Despite the delay, European countries came on board, during March 2020, in placing spatial restrictions to prevent further spread of the disease (9).

Strict spatial regulation has been reinforced by technological advancement in epidemiological tracing using mobile apps. They track interactions between those diagnosed with coronavirus and the people they have come into contact with. The purpose of the apps is to effectively identify those who may have contracted the virus that they may not be aware (11). Many countries worldwide, including Singapore, China, South Korea, Germany, Finland, and Australia, have since developed their tracing app or device. When concerns about personal privacy and state surveillance surface, the governments tend to focus more on technological issues and “brushing them aside as unwarranted or paranoia” (3).

On the other hand, there are demands for deregulation that governs economic aspects of lives. Deregulation is intended to increase economic efficiency, raise productivity, and, ultimately, support jobs and wages. As the below accounts illustrate, governments worldwide have identified that deregulation is essential for the pandemic and the post-COVID-19 recovery. For example, regulatory agencies worldwide have issued expedited processes or streamlined regulations for the industry to set up meetings with the agency during the development process for pandemic-related products. In the US, the transportation department has allowed truck drivers to renew their licenses without following standard procedures if they are directly engaged with emergency relief supplies. The government will introduce a slew of tax incentives to spur corporate investment and reshoring, even allowing big companies to run venture capital business by easing the capital investment restrictions for non-financial entities. According to the policy tracker by the IMF (12), the banks worldwide are providing an extension of loans without additional provisioning or downgrades for borrower's credit status or defer loan installments without penalties to ensure the cash liquidity of businesses. The various governments guarantee new bank loans for businesses to cover operating costs during the pandemic (12). Repayment reliefs on mortgages and personal loans to finance housing were announced worldwide. Some countries waive credit card fees and interests, suspend loan interests payment, and extend tenures of trade instruments (12).

However, as the pandemic becomess an everyday reality, regulatory agencies need to review these potentially conflicting demands in regulation to coherently define their overall regulatory rationale. While the changes during the pandemic may be temporary, policymakers will have to decide whether to keep these changes altogether, or return to pre-COVID-19, or select some of the changes. The choices come with trade-offs of values. For instance, streamlining medical product regulation to promote their access can be beneficial in terms of efficiency, affordability, improved health outcomes, and decreased costs to the health care system overall. However, relaxed privacy or data requirements, less frequent inspections, and less scrutinized safety protocols may risk other public values. How do we balance regulations on the binary scale when the contradictions and complications occur in multiple dimensions? The governance of aggregating and shaping the regulatory changes can be a difficult task under a binary scale. The next section describes a regulatory example of South Korea that describes a precision regulation approach as an alternative way to complement the current binary approach.

## South Korean Approach in Regulation During and Post-COVID-19

South Korea was one of the most severely hit nations in the early days of the COVID-19 outbreak. Following the COVID-19 outbreak, companies in the bio-technology industry were given all the information and support in open competition under emergency fast-tracked approval processes (13). Simultaneous massive public testings reinforced the technologies of biotech companies to attain reliable data, and to improve their inventions. COVID-19 exports of testing kits and personal protection suits increased sharply, uplifting the entire industry and developing treatments, vaccines, and other related areas. The problem-solving nature of regulatory responsesbiotechnology industry in South Korea addressed the regulatory risks in timely fashion to have rapid COVID-19 test kit development.

The Korean Center for Disease Control (KCDC) used emergency fast-track procedures to promote COVID-19 test kits (13). Infectious disease experts in the public and private sectors were called into frequent and urgent task force meetings to devise protocols to engage industry partners in developing the test kits. Appropriate incentives were provided and full transparency of the publicly held data and test methods on the disease. The KCDC considered the fact that initial test kits might not be of high quality, given the limited time for development. The Korean Society for Laboratory Medicine (KSLM) was the key actor in enabling laboratory preparedness and responsiveness for the quality and robustness testing of the test kits. The KSLM also contributed inmaintaining diagnostic testing quality for prototype test kits by providing unbiased validation sites and procedures (14). More than 2,723,960 people had been tested by November 10th (15). This in turn, allowed the biotech industry to share large samples to improve on the test kit accuracy. Korea conducts up to 15,000–20,000 tests a day, with the remainder exported to other countries.

Hence, the precision regulation approach in this case studyhas two critical characteristics: (i) embracing the urgency of the problem in regulations and (ii) involving plural actors to effectively resolve the problem. However, addressing the regulatory problem promptly while upholding quality standards is not an easy task and can be very costly. Indeed, the prerequisites for the precision regulation are likely to be reasonably well-established public health infrastructure, high level of inter-agency trust, and efficient intersectoral communication skills among the policy actors. Those characteristics helped to accelerate the discussions and enable feedback mechanisms to expedite the political processin the South Korean case.

Nonetheless, creating opportunities for discussion and negotiating ways forward has not been the traditional task for policy designers (16). The notion of “polycentric governance” (17), captures an increasingly complex and diversified political landscape in which many actors draw on various forms of material and symbolic power to influence decision-making processes and outcomes. The understanding of polycentricity of the precision regulation approach situates itself in the stream of regulation literature^1^ emerged to focus on innovative approach to achieve compliance, including “responsive regulation” (18), “nodal governance” (19), “steering-at-a-distance” (20), “smart regulation” (21), and “meta-regulation” (22) and Meta-governance (10). The strength of these approaches is that they recognize that the capacity to deliver on regulatory objectives lies primarily with those regulated, rather than those who regulate. The concepts highlight the polycentricity of the regulated actors, contributing to breaking the binary conception of the regulation.

The binary approach to regulation looks at the regulation functioning like a flip switch that turns either on or off (23). This approach may have limited insights into the behavior of the industry actors to the policies and regulations. Suppose, for instance; a dichotomous deregulation approach was taken for the bio-technology industry. In that case, the future regulator has to face the impacts of the changed behavior, practices, and outcomes of the industry actors, which may be low-quality products, moral hazard, and public dependency of the private sector. In the South Korean case, in order to prevent potential pitfalls, precision in regulations was emphasized. The regulatory steps were scrutinized to ensure that the deregulation does not suffer from the future costs for the regulator and the industry. That is the way the government needed to include a variety of actors in the regulatory process. The rapid feedback mechanisms enabled polycentric governance through the regulatory precision approach.

Based on the case study, the precision regulation for the pandemic can be used to reinforce meta-governance with the explicit goal of public value delivery. For the case of deregulation, effective regulation should aim to satisfy political expectations and operational feasibility (24). Precise and targeted deregulation in the bio-technology industry and effective communication in public-private regulatory partnerships have been South Korea's critical enablers of COVID-19 test kit development (13). Furthermore, the industry experts and private sector medical practitioners played crucial parts in testing and validating test kits in the streamlined processes. The sense of urgency to achieve such challenging goals further necessitated the involvement of wide spectrum of actors to join the discussion.

## Conclusion

This article identifies the precision regulation approach, using the case study of biotechnology industry regulation in South Korea. The regulatory approach worldwide is primarily divided between flexible arrangements and deregulation, depending on the sectors and the urgency created by the disease. The article points out that such binary understanding of regulation may fall short of adequately addressing the wide spectrum and the interconnected aftermath of the pandemic. It may be too soon to declare South Korea's regulatory approach as precision regulation because the regulatory responses continue to evolve as the battle against COVID-19 continues. Nonetheless, the South Korean COVID-19 regulatory response on the biotechnology industry can guide other nations struggling to balance the binary scale of regulatory flexibilities. The essence of the South Korean case is the focused attention on the specific problem, striving to incorporate multiple aspects of the problem, and an active engagement between private and public sectors, which can be intuitively applied to various countries. Furthermore, future studies may find more examples of the precision regulatory approach in countries with relatively higher quality of public health infrastructure and high inter-agency trust. It may be timely for scholars worldwide to discuss the new rationale for regulation in post-COVID-19 governance.

## Data Availability Statement

The datasets presented in this study can be found in online repositories. The names of the repository/repositories and accession number(s) can be found in the article/supplementary material.

## Author Contributions

SL and WK have contributed equally to the conceptualization of the article. SL has drafted the article. WK added his insights. SL and WK revised the article together. Both authors contributed to the article and approved the submitted version.

## Conflict of Interest

The authors declare that the research was conducted in the absence of any commercial or financial relationships that could be construed as a potential conflict of interest.

## Footnotes

^1^The literature's primary focus is on regulation in pursuit of public regulatory goals, which will often imply regulation by public regulatory bodies, which may of course involve the mobilization of private actors, civil society, and public-private partnerships.
